# Genome-wide characterization, evolutionary analysis, and expression pattern analysis of the trihelix transcription factor family and gene expression analysis under MeJA treatment in *Panax ginseng*

**DOI:** 10.1186/s12870-023-04390-w

**Published:** 2023-08-01

**Authors:** Jian Hu, Tao Liu, Huimin Huo, Sizhang Liu, Mingming Liu, Chang Liu, Mingzhu Zhao, Kangyu Wang, Yi Wang, Meiping Zhang

**Affiliations:** 1 College of Life Science, Jilin Agricultural University, Changchun, Jilin, 130118 China; 2 Jilin Engineering Research Center Ginseng Genetic Resources Development and Utilization, Changchun, Jilin, 130118 China

**Keywords:** *Panax ginseng*, Trihelix transcription factor family, GT factors, Functional genomics, Methyl jasmonate treatment

## Abstract

*Panax ginseng* is a well-known medicinal plant with several pharmacological uses in China. The trihelix family transcription factors, also known as GT factors, can be involved in the regulation of growth and developmental processes in plants. There have been no in-depth reports or systematic studies about the trihelix transcription factor in ginseng. In this study, the structure, chromosomal localization, gene duplication, phylogeny, functional differentiation, expression patterns and coexpression interactions of trihelix transcripts were analysed using bioinformatics methods based on the ginseng transcriptome database. Thirty-two trihelix transcription factor genes were identified in ginseng, and these genes were alternatively spliced to obtain 218 transcripts. These transcripts were unevenly distributed on different chromosomes of ginseng, and phylogenetic analysis classified the *PgGT* transcripts into five subgroups. Gene Ontology (GO) analysis classified *PgGT* transcripts into eight functional subclasses, indicating that they are functionally diverse. The expression pattern analysis of 218 *PgGT* transcripts revealed that their expression was tissue-specific and spatiotemporally-specific in 14 different tissues of 4-year-old ginseng, 4 different ages of ginseng roots, and 42 farmers’ cultivars of 4-year-old ginseng roots. Despite the differences in the expression patterns of these transcripts, coexpression network analysis revealed that these transcripts could be expressed synergistically in ginseng. In addition, two randomly selected *PgGT* transcripts in each of the five different subfamilies were subjected to methyl jasmonate treatment at different times, and *PgGT* was able to respond to the regulation of methy1 jasmonate. These results provide a theoretical basis and gene resources for an in-depth study of the function of *trihelix* genes in other plants.

**Supplementary Information:**

The online version contains supplementary material available at 10.1186/s12870-023-04390-w.

## Introduction

Transcription factors (TFs) are important regulators that affect growth and development, physiological processes, and secondary metabolism in higher plants; they also influence the regulation of functional genes expression [1]. Under normal conditions, transcription factors with binding function and can be to other proteins domain or DNA sequence promoter region to perform their specific functions [2]. Currently, the GRAS [3, 4], AP2/ERF [5], MYB [6], WRKY [7], NF-Y [8], HD‑Zip [9], bHLH [10], and other specific transcription factor families have been bioinformatically analysed, and their functions have been verified in ginseng. The trihelix transcription factor had been functionally validated in many plants, but no in-depth reports or systematic studies about this gene family in ginseng.

The trihelix transcription factor is one of the first transcription factor families identified in plants. The trihelix transcription factor family is also known as the GT factor family, and its members are composed of three tandem helix (helix-loop-helix-loop-helix) structural domains. This structural domain has binding specificity to the light-response element GT element on the DNA sequence, thereby being classified as a GT factor [11]. The GT factor family has a typical triple-helix DNA binding domain structure that determines the specificity of GT factor binding [12, 13]. The trihelix transcription factor family of *Arabidopsis thaliana* (Arabidopsis), *Nicotiana tabacum* (tobacco), and *Oryza sativa* (rice) are divided into three subfamilies, including GTα, GTβ, and GTγ, which were later classified into five classes, including GT-1, GT-2, SH4, GTγ, and SIPI, according to the amino acids of the functional structural domain. The nomenclature is determined by the first gene discovered [14]. Due to the increasing number of known family members, there are many trihelix TFs whose functions have been validated [13], which include regulation of light-dependent expression, response to biotic and abiotic stresses, and functions involving multiple floral organs, cotyledon morphology, trichomes and embryo development and a range of other developmental processes [15]. In plants, by bioinformatics analysis, trihelix transcription factors have been identified in many species: 29 in Arabidopsis [13], 41 in rice [16], 71 in soybean [17], 36 in tomato [18], 10 in tea [19], 52 in oilseed rape [20], 40 in sorghum [21], and 80 in wheat [22].

Studies in tomato have shown that the ShCIGT transcription factor and SnRK1 interact to increase tolerance to abiotic stresses in transgenic *Solanum lycopersicum *[23]. In Arabidopsis, GT-4 interacts with the TEM2 protein, thereby improving its tolerance to salt stress [24]*.* AtGT2L similarly interacts with Ca^2+^ regulatory proteins, thereby improving resistance to high salt stress. The GT-2A and GT-2B transcription factors of *Glycine max* are transformed into Arabidopsis significantly enhanced salt resistance in transgenic Arabidopsis compared with wild-type plants [25]. Therefore, the GT transcription factor family plays important roles in plant growth and development, biotic and abiotic regulation and salt stress resistance. The GT transcription factor in other plants has been studied, but the study of this transcription factor has not been reported in ginseng.

*Panax ginseng* is a perennial medicinal plant of the genus *Panax* in the family Araliaceae [26, 27]. Recent research has shown that ginsenoside is the main medicinal component of ginseng [28]. At present, cultivation is the main source of ginseng, and the quality of ginseng is affected by internal and external factors such as the culture period length, climate, pests, and diseases [29]. With the increasing demand for ginseng, cultivated ginseng can hardly meet people's demand; therefore, it is important to use modern biotechnological means to improve the content of ginsenosides.

Elicitors refer to factors that have a facilitating effect on the production of target substances, such as secondary metabolites in plant cells, and can be classified as biotic or abiotic according to their nature [3–10]. Since inducers can significantly increase the number of secondary metabolites in plant cells within a short period of time, they are widely studied in secondary metabolite synthesis pathways. Commonly used inducers in ginseng include salicylic acid, jasmonic acid, methyl jasmonate, small molecule compounds, and fungi and their extracts. Methyl jasmonate (MeJA) has become one of the most commonly used inducers in the study of the ginsenoside synthesis pathway due to its good induction effect and stable induction; we selected MeJA as our additional inducer [30].

In this study, the ginseng transcriptome database was used to screen trihelix gene family members and perform systems analysis in terms of structure, function, and expression pattern. The trihelix transcription factor family members of ginseng were found to respond under MeJA treatment of ginseng hairy roots, which will provide valuable data and gene resources for study MeJA response.

## Materials and methods

### Ginseng database

Three ginseng transcriptome databases [31, 32] were constructed in our laboratory and were selected for the main databases for this study. The first transcriptome database was created from 14 tissues of 4-year-old ginseng and consisted of 248,993 transcripts. The 14 tissues were fibrous root, leg root, main root epidermis, main root cortex, rhizome, arm root, stem, petiole, leaflet pedicel, leaf blade, fruit pedicel, fruit pulp, and seed. The second transcriptome database was based on 42 ginseng farmer cultivars of roots collected. The third transcriptome database was obtained from roots of 5-, 12-, 18-, and 25-year-old ginseng roots.

### Identification of *PgGT* genes from the ginseng transcriptome

First, the latest HMM model sequence number (PF13738) of the trihelix transcription factor gene family containing the conserved structural domain of trihelix was used, and related sequences were downloaded from the Pfam database (http://pfam.sanger.ac.uk/, accessed on 1 August 2022) [33]. HMMER 3.0 [34] was used to screen the ginseng transcriptome for subsequent database screening as interrogated sequence one. Afterwards, the Korean ginseng genome was downloaded from the Korean ginseng genome website (http://ginsengdb.snu.ac.kr, accessed on 10 August 2022) to obtain the CDS and protein sequence of trihelix, for subsequent use as the interrogation sequences for the database of the ginseng transcriptome of Jilin ginseng. Finally, nine trihelix family gene sequences that have been validated for function were downloaded from GenBank (https://www.ncbi.nlm.gov/, accessed on 18 August 2022) to use as the interrogation sequences for the ginseng transcriptome from Arabidopsis [13], rice[16], tomato [18] and wheat [22]. Finally, the above sequences were compared and screened in Blastn to remove duplicate sequences, and the correct sequences were screened by the SMART tool online (https://smart.embl-heidelberg.de/, accessed on 24 August 2022) to select transcripts containing the conserved structural domain of trihelix and identify trihelix candidate genes. The sequences were also renamed as ginseng *trihelix* genes, such as *PgGT01*, following the model plant Arabidopsis *trihelix* gene family nomenclature, designating different trihelix genes with a suffix (e.g., *-01*) for different *trihelix* genes.

### Analysis of the physicochemical properties of *PgGT* genes

To further understand the physicochemical properties of PgGT proteins, we predicted and analysed the physicochemical properties of PgGT proteins, such as composition, isoelectric point, and molecular weight, using ProtParam (http://web.expasy.org/protparam/, accessed on 30 August 2022) [35].

### Chromosome localization and collinearity analysis of the *PgGT* gene

The Blastn was used to compare the above identified *trihelix* genes with the above three ginseng genomic datasets to determine their distribution in ginseng chromosomes, localize the *PgGT* gene to ginseng chromosomes [36] and study the collinearity of this gene family in ginseng from Jilin.

### Conserved motif analysis and phylogenetic tree construction of the *PgGT* gene

NCBI ORF Finder (http://www.ncbi.nlm.nih.gov/orffinder/, accessed on 2 September 2022) was used to identify open reading frames of *trihelix* gene family members. Conserved motif analysis was performed using MEME (http://meme.nbcr.net/meme/, accessed on 4 September 2022) [37]. The online Rice Genome Database (http://rice.plantbiology.msu.edu/, accessed on 5 September 2022), Tomato Genome Database (http://solgenomics.net/search/locus, accessed on 5 September 2022), and Arabidopsis Genome Database (https://www.arabidopsis.org/, accessed on 5 September 2022) were searched and protein sequences were downloaded. The protein sequences of the three plants and the protein sequence of PgGT were selected to construct phylogenetic trees with 2000 bootstrap replicates using the maximum likelihood (ML) method by MEGA X software [38] (http://mega.co.nz/). Modifications to the evolutionary tree were visualized using iTOL v5 online software [39].

### GO functional annotation and enrichment analysis of the *PgGT* gene

The *PgGT* transcript function was annotated using Blast2go software (https://www.blast2go.com/free-b2g-trial) [40]. The annotation results were further analysed using GO function annotation.

### Analysis of *PgGT* gene expression patterns

To further analyse the expression pattern of the *PgGT* gene, the Perl software was used to separately identify the expression of *PgGT* transcripts in 42 farmer cultivars of ginseng roots, 14 tissues, and 4 different years of age, containing the complete ORF. Heatmaps were constructed using the R software [41].

### Coexpression network analysis of *PgGT* gene

To further investigate the interaction characteristics between the expression of *PgGT* genes in 42 farmer cultivars, the Spearman correlation coefficients were calculated using SPSS version 23.0. Gene coexpression networks were constructed using BioLayout Express ^3D^ version 3.3 software.

### Expression analysis of the *PgGT* gene under MeJA treatment in ginseng hairy roots

In this study, the ginseng cultivar of Damaya from Jilin was used as material for germinating root induction [42]. First, ginseng seeds were cleaned and sterilized, germinated on 1/2 MS medium and incubated at 23 °C for 15 days until the seedling leaves were fully expanded. The seedlings were cut into small segments of approximately 0.5 cm, transformed by infestation with *Agrobacterium germinatum* strain C58C1 and cultured at 23 °C in the dark, and eventually germinating roots were obtained approximately one month after passing the screening. Each induced single ginseng hairy root was further cultured separately to ensure homogeneity of the genetic background. The screened ginseng hairy roots were cultured in shake flasks containing 150 mL of 1/2 MS liquid medium with an initial inoculum of 1.0 g, inoculation temperature of 22 °C and shaker speed of 110 rpm for 23 days.

Ginseng hairy roots were treated under 200 μM concentrations of MeJA, previously validated as optimal in the laboratory, and collected at 0 h, 6 h, 12 h, 24 h, 36 h, 48 h, 60 h, 72 h, 84 h, and 96 h after treatment. Three biological replicate samples were collected at each time point. Two *PgGT* transcripts from each of the five families of ginseng *trihelix* genes were subjected to methyl jasmonate treatment. The *Actin 1* gene was used as an internal reference gene in fluorescent quantitative RT-PCR. SH4 subfamily: *PgGT24-01* and *PgGT24-03*, GT-2 subfamily: *PgGT03-03* and *PgGT18-02*, GT-1 subfamily: *PgGT19* and *PgGT23-12*, GT-γ subfamily: *PgGT20-05* and *PgGT20-06*, SIP I subfamily *PgGT09* and *PgGT16-02*. The cDNA from ginseng hairy roots treated under MeJA was used as templates, and the fluorescent quantitative RT-PCR system using SYBR premix EX Taq II (Takara, Dalian, China) included 5.0 μL SYBR premix Ex Taq II, 0.2 μL ROX II, 0.4 μL design primer, 0.5 μL cDNA, and 3.5 μL ribonuclease-free water. Reactions were as follows: predenaturation at 95 °C for 30 s; PCR at 95 °C for 5 s and 60 °C for 34 s for 40 cycles; melting at 95 °C for 15 s, 60 °C for 60 s, and 95 °C for 15s. The 2^−ΔΔCt^ method was used to analyse the fluorescent quantitative RT-PCR results in this study [43].

## Results

### Screening of the whole transcriptome *PgGT* gene and analysis of its physicochemical properties

Ginseng transcripts were screened by Blast's comparative screening, combined with the Pfam database using online HMMER software, SMART and other databases, and transcripts with complete structural domains of trihelix were retained. Finally, 218 transcripts from *PgGT-01* to *PgGT-32* were obtained under 32 gene IDs (Table S1).

To investigate the sequence profile of the GT transcription factor of ginseng, the sequence length of the *PgGT* gene was statistically calculated to be between 298 bp (*PgGT30*) and 4883 bp (*PgGT25-07*). The physicochemical properties of PgGT proteins were used to analyse by the online software ExPASy, and the longest corresponding protein consisted of 849 amino acid residues, with molecular weights ranging from 5997.72 kD (PgGT32) to 94,585.65 kD (PgGT10) and isoelectric points (pI) of 4.54 (PgGT09) to 10.66 (PgGT14-10 and PgGT14-11) (Table S2). We determined the distribution of the mean hydrophilic values of PgGT, and most of the PgGT proteins were hydrophilic. From the results, some of the PgGT proteins under the same gene ID also had different physicochemical properties, and the PgGT proteins under different gene IDs differed greatly in physical and chemical properties (Table S2), indicating that different genes play different roles.

### Chromosome localization and collinearity analysis of the *PgGT* gene

**Fig. 1:**
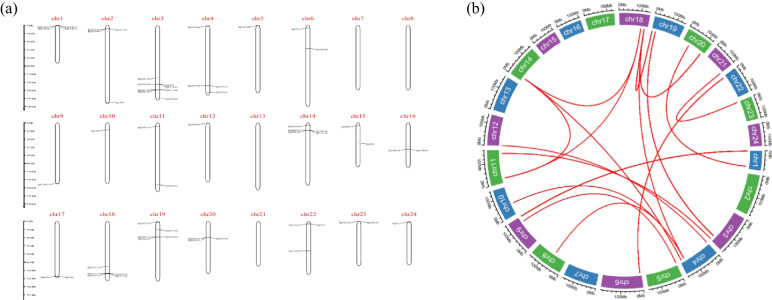
*PgGT* chromosome distribution and covariance analysis. **a** Distribution of ginseng GT family members on ginseng chromosomes. **b** Covariance analysis of *GT* gene family members in ginseng chromosomes, The red line represents tandem duplication of the same gene on different chromosomes

The *PgGT* genes were unevenly distributed on 20 of the 24 chromosomes of ginseng, as shown in Fig. 1a. Four chromosomes, chr7, chr8, chr13, and chr21, did not contain *PgGT* genes*,* and chromosome chr3 contained the highest number of *PgGT* genes. By intraspecific colinear analysis [44], there were 45 pairs of colinear trihelix genes, as shown in Fig. 1b (Table S3). Members of the ginseng trihelix family may have increased due to gene duplication. The clustering or scattering of genes on chromosomes occurs due to gene duplication.

### Conserved motif analysis and evolutionary analysis of *PgGT* gene

To determine the accuracy of the *trihelix* gene again, the prediction of motifs for 218 *PgGT* genes, 20 motifs were selected for analysis, and motif1 and motif2 were found to be present in most of the family members. These motifs were presumed to be part of the conserved structural domain of the *PgGT* base. The analysis revealed that the trihelix family proteins have invariant or identical structural domains, and they generally have important functions that cannot be altered. These motifs contain a minimum of 21 amino acids and a maximum of 50 amino acids. The same family sequences exhibited similar motif characteristics, further demonstrating the stability of the conserved structural domains of *PgGT* genes.

**Fig. 2:**
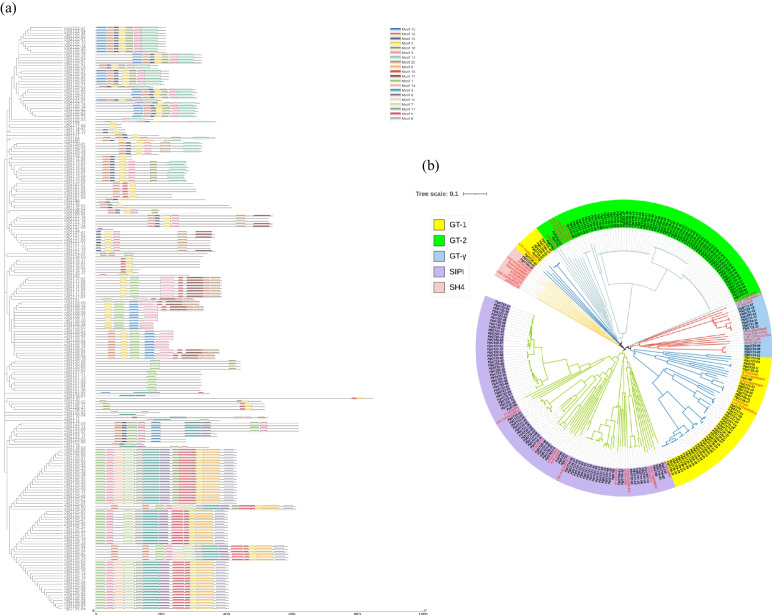
Phylogenetic relationships and conserved motif analysis of PgGT proteins. **a** Neighbor-joining phylogenetic tree of PgGT (1000 replicates of self-help values). Distribution of conserved motifs in PgGT proteins. Colored boxes represent different patterns. The length of the boxes represents the length of the patterns. **b** Trees were constructed using MEGA X and neighbor joining methods (1000 replicates of bootstrap values). Colored branches indicate different subgroups. Red font represents outgroup Trihelix proteins. Black font represents PgGT proteins

The conserved motif distribution of the trihelix transcription factor of ginseng remained largely consistent with the branching sequences of the phylogenetic tree. The PgGT protein sequences were classified into five subfamilies, including GT-1, GT-2, GTγ, SIP1, and SH4, by comparison with protein sequences from Arabidopsis, tomato, and rice (Fig. 2), with the tandem repeat structural domain of the triple helix hydrophobic core containing two tryptophan residues, while the third residue involved in core formation was a tryptophan or phenylalanine. According to Luo et al. [45], triple helix genes with phenylalanine as the third residue belong to the GT-2 and GTγ subfamilies. Those containing the third isoleucine residue are members of the SIP1 subfamily. In the phylogenetic tree of *G. japonica*, two of these subfamilies, GT-2 and SIP1, accounted for the largest proportion of the tree and contained the largest number of transcripts. The SIP1 subfamily contains 80 transcripts, such as *PgGT22-01*, *PgGT04*, *PgGT13-01*, etc. All contain motifs 1, 2, 3, and 12, and individually, motifs 14 and 19, accounting for 36.7% of the total *PgGT* transcripts. The GT-2 subfamily contains 75 *PgGT* transcripts, which accounts for 34.4% of the total PgGT transcripts. The GT-1 subfamily contains 49 transcripts, such as *PgGT01*, *PgGT02*, *PgGT28-0*1*,* etc., which contain motifs 9, 13, 14. 22.4% of the total *PgGT* transcripts. The GT-γ subfamily contains 12 transcripts, mainly containing motif 1. 5.5% of the total *PgGT* transcripts. The SH4 subfamily contains only two transcripts, *PgGT24-01* and *PgGT24-03,* and contains only motif 1, motif 12 and motif 18, which accounted for 0.9% of the total *PgGT* transcripts*.* In terms of affinity, the GT-2 subfamily and GT-1 subfamily were more closely related. As shown in Fig. 2, different transcripts were clustered together, indicating the same or similar functional expression between them.

### GO functional annotation of the *GT* gene in ginseng

**Fig. 3:**
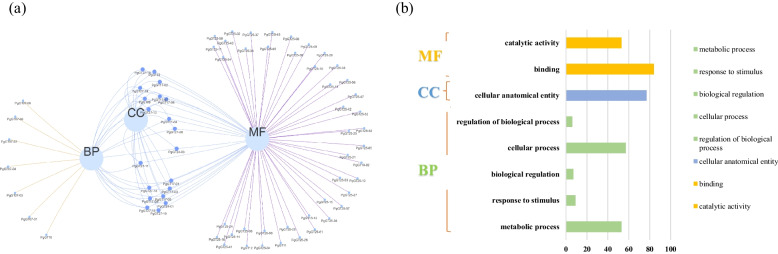
Functional categorization of the *PgGT* genes by gene ontology (GO). **a** Gene Ontology (GO) Venn diagram of PgGT protein. **b** Visualization shows 8 GO terms at the molecular function (MF), biological process (BP) and cellular component (CC) level 2. The x-axis shows the number of abundant proteins and the y-axis shows the information of GO terms

We functionally annotated 218 transcripts using the Blast2go software, and identified GO function of the trihelix transcription factor family members from the annotation information, and generated a Venn diagram of the results (Fig. 3a, Table S4). These 218 transcripts were mainly annotated to 3 major GO functional categories: biological processes (BP), molecular functions (MF), and cellular components (CC). At level 2, they are divided into 8 subcategories (Fig. 3b). BP includes biological regulation (GO:0065007), cellular process (GO:0009987), response to stimulus (GO. 0050896), and stimulation (GO:0050897): response to stimulus (GO:0050896), regulation of biological process (GO:0009987), metabolic process (GO:0008152):CC contains cellular anatomical entity (GO:0110165). MF contains binding function (GO:0005488) and catalytic activity (GO:0003824). At level 2, only 56 genes have a single function. To obtain more information, the eight GO functions annotated to *PgGT* at the level 2 level were analysed by computational statistics based on the annotation information of 248,993 unigene sequences from Jilin ginseng as a reference. The results overwhelmingly shown that the *PgGT* gene family members have a binding function to DNA, are transcriptionally active and regulated, and play an important role in the growth and metabolism in ginseng.

### Analysis of *PgGT* gene expression patterns

**Fig. 4:**
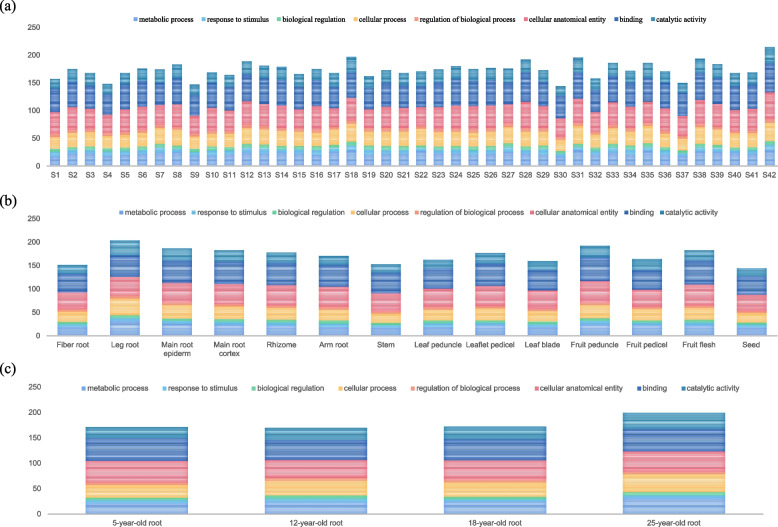
Variation in the number of transcripts of *PgGT* in functional categories in 8 GO functional terms. **a** 42 different farmers cultivars. **b** 14 different tissues of 4-year-old ginseng plants. **c** 4 different ages

**Fig. 5:**
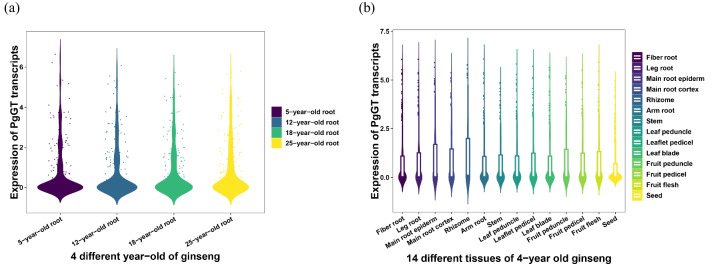
The expression pattern of *PgGT* genes in *Panax ginseng*. **a** The violinplot of all *PgGT* genes expression in different tissues of ginseng. **b** The violinplot of all *PgGT* genes expression in different ages roots of ginseng

To allow a clear analysis of the expression of *PgGT* transcripts in ginseng, we extracted the expression of the above transcripts in 42 farmers cultivars of ginseng roots (Fig. 4a), 14 different tissues of 4-year-old ginseng (Fig. 4b), and 4 different of ages (Fig. 4c) and analysed them statistically. Among the 42 farmers cultivars, the farmers S42 had the highest number of transcripts with 214. S28 had the lowest number with only 169 transcripts. The number of gene transcripts was higher in the branched roots and fruit stalks among the 14 different tissue parts of the 4-year-old ginseng. The 25-year-old ginseng root was annotated with the highest number of functional transcripts among the four different annual roots. These results suggest that there are some differences in the functions of the analysed *PgGT* transcripts, but their functions are largely consistent across genotypes, tissues, or developmental stages of expression. Validation of their tissue-specific and spatiotemporal specificity was carried to further investigate whether *PgGT* transcripts have a regulatory function. To better understand the expression level of *PgGT* transcripts in 14 different tissue parts and 4 different ages of ginseng, violin maps were drawn for 14 tissue parts and 4 different ages (Fig. 5). The results showed that the expression of *PgGT* transcripts was low in stems and seeds and high in roots; the expression of *PgGT* transcripts was relatively consistent and concentrated in the four different ages, with relatively low expression. The analysis of *PgGT* transcript expression profiles of Jilin ginseng revealed that the expression of *PgGT* transcripts was influenced by the tissue type and different annual growth in ginseng.

**Fig. 6:**
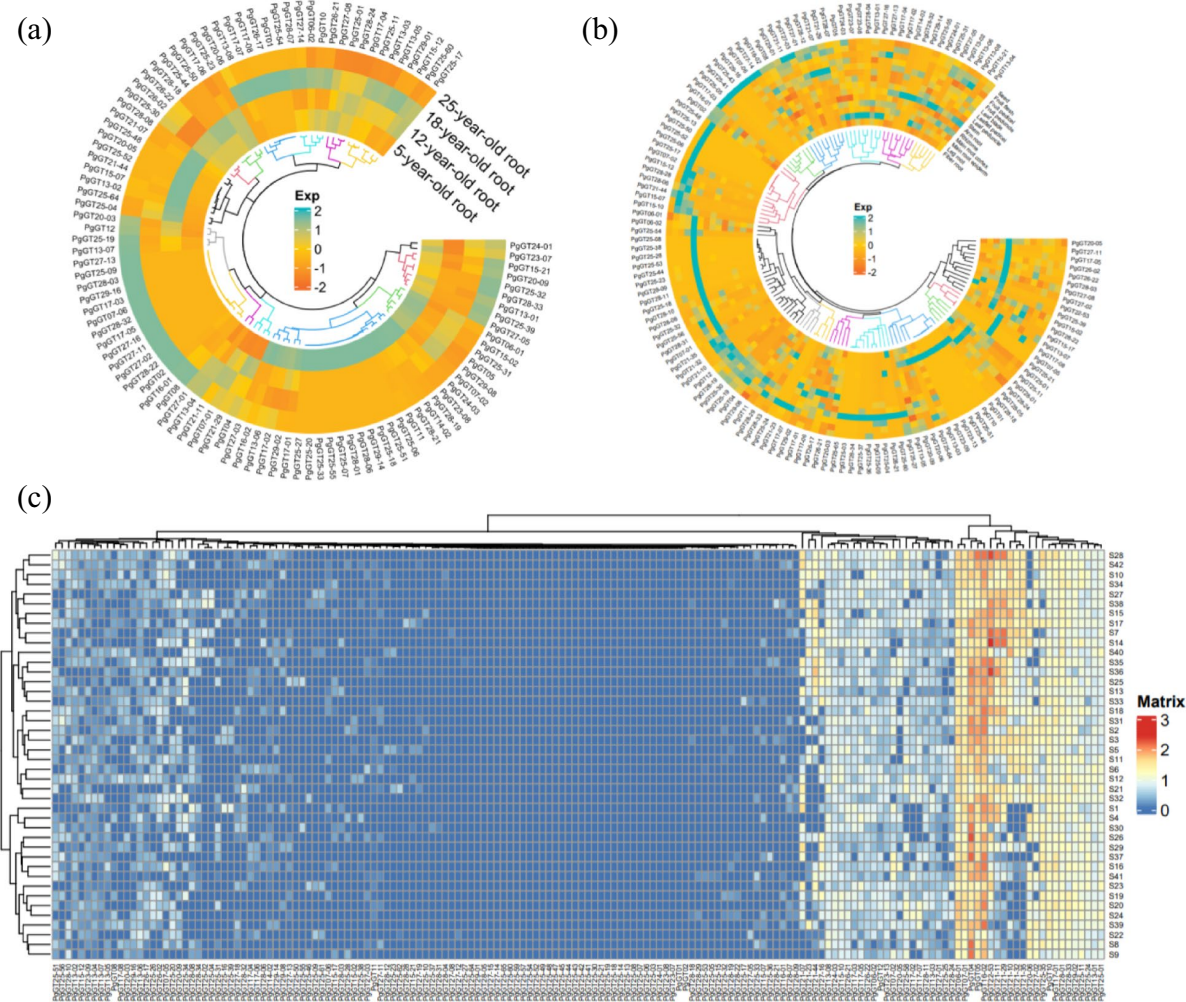
Heatmaps analysis spatiotemporal expression patterns of *PgGT* transcripts in *Panax ginseng*. **a** The *PgGT* genes expressed in the 4 different ages (5, 12, 18, 25 years) of ginseng roots. **b** The *PgGT* genes expressed in the 14 different tissues of 4-year-old ginseng. **c** The *PgGT* genes expressed in the 42 farm cultivars of 4-year-old ginseng roots

Heatmapping of the expression of 162 *PgGT* transcripts with complete ORFs showed that 64.8% of the transcripts were expressed in four different annual roots, and 35.2% of all transcripts were not expressed. In these 106 expressed transcripts, the expression of some transcripts increased with time. Expression was significantly different at different developmental time stages (Fig. 6a). Most of the transcripts were expressed in different tissues, especially in root tissues (Fig. 6b). Among the 42 farmers’ cultivars, each transcript was differentially expressed; for example, *PgGT-04*, *PgGT-05*, and *PgGT16-02* were highly expressed in 42 cultivars with high expression (Fig. 6c). The same gene had different expression in different transcripts, different times, different tissues, and different cultivars, but some transcripts also had the same or similar expression activity pattern in time and space, showing the phenomenon of coexpression. This indicates that *PgGT* transcripts have coexpression patterns among themselves, which are diverse and complex with spatial and temporal specificity.

### Coexpression network analysis of *PgGT* genes

**Fig. 7:**
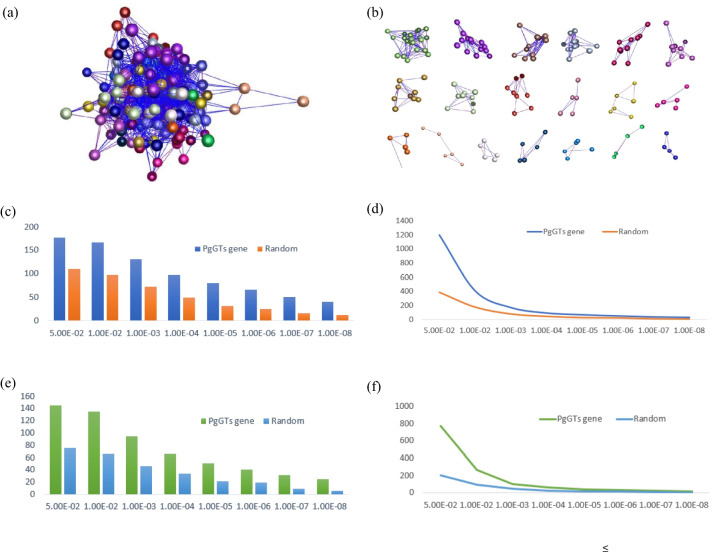
Co-expression network of *PgGT* genes expressed in 42 farming cultivars. **a** Co-expression network of 218 *PgGT* genes constructed at *P* ≤ 5.0E-02. It contains 177 gene transcription nodes and 1197 interaction edges. **b** The 19 clusters of this network. **c**, **d** Network formation trends at different *P* values: variation in the number of nodes (**c**) and edges (**d**). **e**, **f** 2/3 of *PgGT* genes statistics of network formation trends in the number of edges (**e**) and nodes (**f**). Ginseng unknown gene transcripts from the first database were randomly selected as controls

To validate genes with related functions in the *PgGT* gene family, we performed a coexpression network analysis of the expression of *PgGT* transcripts from 42 different farmer cultivars. The same unknown number of ginseng transcripts randomly selected from the database was used as a reference control. Among the 4-year-old roots of the 42 genotyped cultivars, 177 of the 218 *PgGT* transcripts tended to be expressed phase-linked, forming a reciprocal network of 177 nodes with 1197 edges on *P* ≤ 5.0E-02 (Fig. 7a). A total of 177 transcripts were divided into 19 classes (Fig. 7b). The remaining 41 were not in the network because they were silent and not expressed in the roots of 42 genotypes. A comparison with randomly selected unknown ginseng transcripts showed that *PgGT* transcripts were more likely to form a coexpression network at different *P* values from 5.0E-02 to 1.0E-08, indicating that they have more nodes and edges (Fig. 7c, d). Two-thirds of the data were selected to determine the viability of these data (Fig. 7e, f).

### *PgGT* gene expression regularity under MeJA treatment

**Fig. 8:**
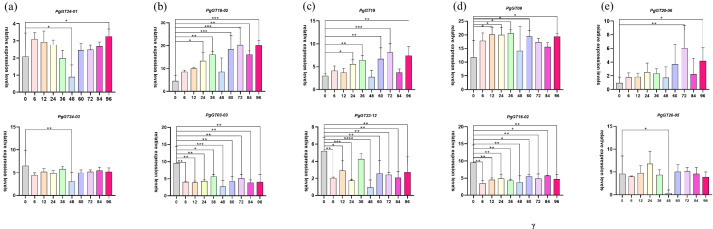
Response of the *PgGT* gene family under MeJA treatment. **a**-**e** The relative expression of *PgGT *transcripts in SH4, GT-2, GT-1, SIP I, and GT-γ isoforms was observed in roots treated under MeJA. Differences in the expression of *PgGT* genes between control and MeJA treatment the ginseng hairy roots were analyzed by ANOVA. X shows the time (h) of MeJA treatment the ginseng hairy roots, Y represents the relative expression levels of genes in the hairy roots of ginseng

In this study, the effect of different times under MeJA treatment of the *PgGT* gene on ginseng hairy roots was investigated in untreated control and MeJA-treated ginseng hairy roots compared with the control. The results shown (Fig. 8) that two *PgGT* genes were regulated for each subtype, and five *PgGT* genes, *PgGT24-03*, *PgGT03-03*, *PgGT23-12*, *PgGT16-02*, and *PgGT20-05,* were upregulated after the addition of MeJA regulation after MeJA treatment. The five *PgGT* genes *PgGT24-03*, *PgGT03-03*, *PgGT23-12*, *PgGT16-02*, and *PgGT20-05* showed a downregulation trend, for example, *PgGT20-05* was downregulated 15-fold at 48 h; in contrast, the other five *PgGT* genes were downregulated. For example, in the SH4 subfamily, *PgGT24-01* was significantly upregulated at 48 h and 96 h, but *PgGT24-03* was the opposite and highly significantly downregulated at 48 h; in the GT-2 subfamily, *PgGT03-03* was highly significantly downregulated at all time periods except 36 h, *PgGT18-02* was significantly upregulated at 24 h and highly significantly upregulated at 36 h, 72 h, 84 h, and 96 h were highly significantly upregulated; in the GT-1 subfamily, *PgGT19* had similar results to *PgGT18-02,* but *PgGT23-12* was significantly downregulated at all time periods except 36 h; in the GT-γ subfamily, *PgGT20-05* was significantly downregulated at 48 h*, PgGT20-06* was highly significantly upregulated at 72 h and significantly upregulated at 96 h; and in the SIP I subfamily, *PgGT09* was significantly downregulated at 12 h, 24 h, 36 h, 60 h, and 96 h significantly upregulated, and *PgGT16-02* showed downregulation at all time periods. Therefore, it can be concluded from the results that *PgGT* genes are influenced by the regulation of MeJA treatment.

## Discussion

In contrast to the well-known transcription factor families of GRAS, MYB, WRKY and NAC, *GT* gene family had received attention only in recent years and was first identified in the nucleus of pea. *GT* gene family is a small family of transcription factors and the number of genes were first identified by its involvement in the light response, then analysed in different plants and found to play an important role in the regulation of plant growth and development and response to stress [46]. Within this study, multiple ginseng databases and multiple screening methods were utilized to concatenate multiple results, which improved the accuracy of screening and made the screening results more realistic and reliable than following a single screening method.

A final screen of 218 *PgGT* transcripts from 32 *PgGT* genes was performed using bioinformatic methods. The number of genes in this family exceeds that of plants such as Arabidopsis [13], rice [16], and wheat [22], and the possible reason for this is thought to be related to the fact that ginseng is an allotetraploid plant. In diploid plants, the number of trihelix genes ranges from 10 to 80, with little difference in the number of the *trihelix* genes between families and genera [13, 16–22], while the number of trihelix genes in heterotetraploid ginseng is higher than that in other species. By collating and counting the physicochemical properties of the proteins of the ginseng trihelix transcription factor family members, differences were found in the molecular weight, pI values, and instability indices of the proteins. These results also demonstrate differences in the function of the *PgGT* transcripts. Different functions are also exhibited under *PgGT* gene with the same ID. Additionally, factors that affect gene number and function, for example, polyploidy and segmental and tandem duplication, could also extend the gene number of plant gene families; in the case of mutations in the structural regions of the gene, the new members probably show different expression patterns [47, 48].

The chromosome localization results show that the sequences are not equally distributed on the 24 chromosomes, and four chromosomes do not have *PgGT* genes because some fragments could not be mounted on the chromosomes during genome assembly; therefore, some fragments remain in the reference genome in addition to the chromosomes. For intraspecific codomain analysis, there were 45 pairs of codominant *GT* genes in ginseng. Following the occurrence of WGD in the common ancestor of all extant angiosperms, ancient WGD has been associated with increased rates of species diversification in some cases. Polyploidy or WGD is a common feature of plant genomes and contributes to changes in genome size and gene content [49].

Conserved structural domains have important functions that cannot be altered and are central to genes. By structural domains, we mean independent regions of stable structure in proteins that are made up of combinations of different secondary and supersecondary structures. Structural domains are also functional units of proteins. In multistructural domain proteins, different structural domains are often associated with different functions. The finding that conserved motifs exhibited in the same subfamily are identical or different demonstrates the stability and conservation of the family, and it is hypothesized that the different expression of different subfamilies may be caused by differences in conserved motifs and structures. The distribution of motifs indicates that genes containing identical motifs are likely to be generated by amplification of genes within the same group (Fig. 2a). A comparative analysis with other plants classified the *trihelix* gene family into five classes in ginseng (Fig. 2b), similar to the evolutionary classification of GT in Arabidopsis, rice, and tomato, which is consistent with the results of previous studies. The clustering in ginseng, tomato, Arabidopsis, and rice could indicate that the origin of the *GT* gene family in ginseng can be traced back to before the divergence of monocotyledons and dicotyledons. Moreover, we observed that ginseng is more closely related to Arabidopsis evolution than many other plants [50]. This can be explained by the higher similarity between the *GT* gene family of ginseng as a dicotyledonous plant and the *GT* gene family of Arabidopsis, which is also a dicotyledonous plant, and the higher conserved nature of the *GT* gene family members in ginseng.

The *GT* gene family members had been reported in biological processes such as growth and development. They were functionally classified by GO functional analysis into eight categories, including biological regulation, cellular processes, stimulus response, biological process regulation, metabolism, cellular anatomical entities, binding function, and catalytic activity. Among them, most *GT* genes were annotated to binding function and catalytic function. It is suggested that *PgGT* genes can function as transcription factors mainly by binding to the promoter regions of genes, and different *GT* genes can perform synergistic or resistance functions in specific biological processes in ginseng.

The *GT* gene family is widely expressed in different species at different tissues and developmental times, and there are differences in expression patterns. Arabidopsis *ASI1* is a negative regulator of the seedling embryonic period and maintains control of seed filling time. *AT5G03680* Arabidopsis *PTL* (*PETAL LOSS*) can inhibit the growth of sepal size, and loss of its gene can lead to a reduction in petal number [51]. In tomato, *SIGT11* plays a role in the typing of floral organs, and overexpression of the *SIGT-1* transcription factor in transgenic tomatoes promotes shorter internodes and smaller leaves in plants [52]. Whether the tightness between *PgGT* genes will make them interact with each other needs to be proven by further experiments. Based on the constructed relationship diagram of network interactions (Fig. 7), different *PgGT* genes have different functions, which is consistent with the results of the functional analysis above.

In the expression analysis, *PgGT* genes were equally widely expressed in 42 farm cultivars of ginseng, 14 tissues and 4 ages of roots. *PgGT* genes were significantly expressed in the roots, performing multiple different functions with spatial and temporal specificity. By network analysis, *PgGT* gene family members formed a coexpression network, and the family members functioned as genes in a synergistic manner. Gene heatmap analysis of *PgGT* genes indicated that the coexpression of these genes was attributed to functional similarity, which was synergistically regulated and thus exercised functions. For example, Arabidopsis trihelix transcription factors AT3G11100 (VFP3) and At5g05550 (VFP5) interact with the protein VirF in *Agrobacterium virulence* [53]. Whether the tightness between *PgGT* genes will allow interaction between them needs to be proven by further experiments in ginseng.

Most *PgGT* genes changed at different time points after MeJA treatment, especially the GT-2 family, which showed significant up- or downregulation at each time point. The use of small molecule compounds such as methyl jasmonate as inducers has become a popular approach to stimulate the synthesis of many commercially valuable secondary metabolites in plants. The relative expression of the *PgGT* gene was changed by treating ginseng hairy roots with methyl jasmonate. These results suggest that GT transcription factors are responsive to MeJA treatment, and these studies can identify candidate genes for rapid response to MeJA for in-depth mechanistic studies of GT transcription factor response and thus explore changes in response to biological phenotypes.

## Conclusion

In recent years, with the convergence of cell engineering, bioinformatics, genomics, metabolomics and other related disciplines, it has become important to use cell factories and synthetic biology to regulate plants at the molecular level. In this study, 218 *PgGT* transcripts were screened by system bioinformatics, and basic information such as chromosomal localization was obtained. GO functional annotation showed that the 218 annotated *PgGT* transcript were classified into eight subclasses according to function. The *trihelix* gene family members of ginseng were classified into five classes by sequence comparison of plant proteins from different species. The expression analysis results showed that *PgGT* was found in different families at different times. The results of expression analysis shown that *PgGT* has different expression patterns in different families at different ages and in different tissues and is widely involved in the growth and developmental regulation of ginseng. *PgGT* genes form a tight coexpression network and work together. We found that most *PgGT* genes responded to the regulation of methyl jasmonate through MeJA treatment experiments*.*

## Supplementary Information

**Additional file 1: Table S1. **Basic information of *PgGT* gene family.

**Additional file 2: Table S2.** PgGT proteins under different gene IDs differ greatly in physical and chemical properties.

**Additional file 3: Table S3.** Chromosome localization and collinearity analysis data of *PgGT* gene.

**Additional file 4: Table S4.** The classification, annotation and GO functional categorization of the *PgGT* gene transcripts.

## Data Availability

All data analysed during this study are included in the supplementary information files, and RNA-seq data have been deposited in the Sequence Read Archive (SRA) (https://www.ncbi.nlm.nih.gov/sra) to NCBI under BioProject PRJNA302556 (SRP066368 and SRR13131364-SRR13131405).
